# The effect of life coaching on psychological distress among dental students: interventional study

**DOI:** 10.1186/s40359-020-00475-5

**Published:** 2020-10-14

**Authors:** Khalid Aboalshamat, Duha Al-Zaidi, Duha Jawa, Hanouf Al-Harbi, Raghad Alharbi, Shahad Al-Otaibi

**Affiliations:** 1 Dental Public Health Division, Preventative Dentistry Department, College of Dentistry, Umm Al-Qura University, Makkah, Saudi Arabia; 2 Dental Intern, College of Dentistry, Umm Al-Qura University, Makkah, Saudi Arabia

**Keywords:** Life coaching, Quasi-experiment, Saudi Arabia, Dental students, Psychological well-being, Depression, Anxiety, Stress, Resilience

## Abstract

**Background:**

Depression, stress, and anxiety are common psychological conditions among dental students in many countries around the world. A number of researchers have found life coaching to be effective at reducing psychological distress. The aim of this study was to assess the effect of a life coaching program on dental students’ psychological status.

**Methods:**

A quasi-experiment study with two arms was conducted on 88 female dental students at Umm Al-Qura University (study group = 44; control group = 44). The psychological status was assessed by questionnaire before and after intervention. The questionnaire was composed of the Depression and Anxiety Stress Scale (DASS-21), Resilience Scale (RS-14), the Psychological Well-Being Scale–Short (PWB-S), and goal approach questions. The study group received a coaching program comprising one lecture for 1 h and five phone coaching sessions over 5 weeks, while the control group received no intervention.

**Results:**

The study group showed a significant reduction in depression, anxiety, stress, resilience, and self-acceptance according to the PWB-S scale. Also, goal approach was significantly improved. On the other hand, the control group showed a significant reduction on the RS-14 only. The differences in the tested scales between the study group and the control group from pre-intervention (T1) to post-intervention (T2) showed significant differences in depression, stress, self-acceptance, and goal approach measurements per t-test.

**Conclusion:**

The study’s findings showed that life coaching had the effect of reducing psychological distress, which encouraged the implementation of coaching practice in the daily life of dental students.

## Background

High levels of distress among dental students has been reported in several systematic reviews [1, 2] that showed the prevalence of distress varied from one country to another and for different psychological constructs. For example, the prevalence of depression ranged from 2.8 to 41%, anxiety ranged from 47 to 67%, and stress ranged from 70 to 72% [3–6]. This is also true in Saudi Arabia, where two studies conducted in different cities found the prevalence of depression to range from 67.4% to 69.9%, anxiety from 66.4% to 79.7%, and stress from 64 to 70% [7, 8]. In fact, dental students face many stressors, including frequent exams, clinical cases needed to be finished each year, time constraints, anxious patients, and possible conflicts with colleagues and staff [1, 2]. This psychological distress was found to be associated with decreases in students’ productivity, work quality, and life satisfaction and with increases in health problems and poor academic performance [1, 2, 9, 10]. This implies a high burden from such phenomena and justifies the need to intervene with solutions to help dental students [11, 12].

In fact, life coaching programs can be used for this purpose and have been used to improve psychological well-being in previous studies [13, 14]. Life coach has several definition, and one of the well cited article defined life coaching as “a collaborative solution focused, result-orientated and systematic process in which the coach facilitates the enhancement of life experience and goal attainment in the personal and/or professional life of normal, nonclinical clients” [15]. Several studies have suggested that coaching has a positive effect on many aspects of life, including workplace performance, health behaviors, goal-setting skills, and minimizing of chronic illness [16–18].

Among medical physicians and students, there were positive results from the efficacy of coaching for reducing psychological distress. For example, a pilot qualitative interventional study in the United States used well-being coaching for 11 physicians and found that there was an improvement in the physicians’ resilience along with a reduction of stress [19]. Further, a three-armed randomized controlled trial (RCT) with medical students in Germany found a small but significant improvement in stress for groups who received short-term coaching sessions as compared to the waiting (control) group [20].

With regard to dental practitioners and students, there has been only one interventional study that assessed coaching for dental students [11]. This study used a self-development coaching program for dental students at Umm Al-Qura University in Saudi Arabia and was aimed at reducing levels of depression, anxiety, and stress, in addition to improving academic performance as compared with a placebo control group. The study results indicated significant short-term improvement in depression and anxiety; however, a relapse was noted after one month, indicating no significant effect in comparison to the control group [11]. However, that study used a self-development coaching program, which is different from life coaching [21]. In addition, this was a short intervention where the program was only a two-day workshop.

Bearing in mind the limitations of the prior coaching intervention studies and the scarcity of studies within the dental profession, the aim of this study was to evaluate the effects of a life coaching program on dental students’ psychological health. The hypothesis of this study in alternative format (H1) is: there is no association between life coaching and psychological distress (depression, anxiety, stress, resilience, autonomy, environmental mastery, personal growth, positive relations with others, purpose in life, and self-acceptance) and goal approach.

## Methods

### Participants

This was a quasi-experiment study with a study group (SG) and a control group (CG). The participants were female dental students (for bachelor degree of dental surgery) at Umm Al-Qura University (UQU), Makkah, Saudi Arabia, during the 2018–2019 academic year. Invitations were sent to 154 students, who were enrolled according to their willingness to participate in the SG or the CG. Participants’ inclusion criteria included being a female dental student at UQU (identified by their academic card) and being in the second through sixth year at that time. The exclusion criteria included any participants receiving psychological treatments or medications, those who did not attend the study introductory lecture, and/or participants who did not sign the consent form.

**Fig. 1:**
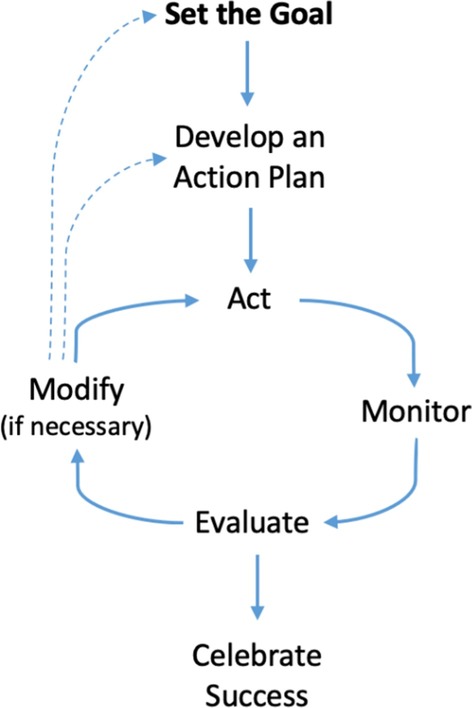
Cycle of self-regulation [22]

The recruiting was conducted by personal invitation from the research team to female class leaders for each academic year. This invitation was given in the first week of the second term and included a research information sheet, consent form, and baseline questionnaire (T1). All participants were required to attend a lecture about the study, the meaning of the coaching, and the process, which was conducted in the second week of the second term during a break from classes in a lecture room in the Faculty of Dentistry at UQU. The lecture explained the concept of solution-focused coaching, stages of change, the cycle of self-regulation change (Fig. 1) [22], creating dreams, and finally, goal setting and making specific plans. These topics were taken from a coaching book [23]. The lecture was given by an expert coach with more than 10 years of experience in life coaching, coaching, and self-development training field. He also has a PhD degree with a thesis in self-development coaching, and professional certificate in training and assessment (that included module of coaching) from The University of Queensland.

After the lecture, each participant was given the choice to participate in the SG or CG. All students in both groups were required to sign the consent form and answer the T1 questionnaire. Participants were free to withdraw from the study at any time with no consequences, which was explained on the study consent form. A pilot study was conducted using 10 senior students for two weeks to test the intervention conduction, process and the questionnaire answering for understanding, syntax, organization, and flow. The participants data of the pilot study were not included in the main study results.

### The intervention: life coaching program

The intervention (independent variable) consisted of attending focused solution-based life coaching program sessions via phone, derived from book *Coach Yourself* [23]. The life coaching intervention comprised five one-on-one phone coaching sessions at the beginning of each week, lasting 15 min and at a convenient free time for participants. Coaching started in the third week of the second term of the 2018–2019 academic year.

The participants in SG were coached by five senior dental students who had received intensive coaching training for one month by the expert coach, and then each one of them practiced to do life coaching for 15 sessions under the supervision and feedback of the expert coach. The coaching was not mutual, and coaching sessions were focused only on coachees. It should be mentioned that they were not fully licensed to be professional life coach by a formal organization. Each participant was assigned to one of the five coaches in a random manner. The phone coaching sessions were standardized using the GROW model [24], which is based mainly on asking consecutive questions to help the participant reach their desired outcomes [25]. GROW stands for goals, reality, outcomes, and wrap-up, as detailed in Table 1. This model aims to help participants establish a solution-focused systematic approach through the self-regulation cycle of monitoring and evaluating progress toward their goals. All participants in both groups were asked to select a goal they wanted to achieve by the end of the intervention period (5 weeks). The participants in the CG received no coaching or intervention during this time.

**Table 1:** GROW model questions [25]

Acronym term	Description	Example questions
Goal	The role of the coach is to help the participant identify their goals (SMART) during the session	What do you want to achieve in this session? How do you want to feel afterward?
Reality	Examine the reality of their goals Participant awareness of their present situation	What has happened during the past week? Did you encounter any problems in trying to achieve your targets? How did you handle the problems?
Options	Look at and assess the available options Guided to use solution-focused and action-oriented thinking and brainstorming	What is the full range of possible actions in these circumstances? What are the costs and benefits of taking a particular action?
Wrap-up	Draw up a specific action plan Give the participant direction for what they should do next	What will you do if you find these things are getting in the way? List some specific tasks and people who can support you

### Data collection and measurement outcomes

Data were collected from participants twice using the study questionnaire at the second week of the second term (the start of the study; T1) and at the seventh week of the second term, after participants received the fifth and final coaching phone session (T2). The questionnaires used at T1 and T2 were identical and took about 5 to 10 min to answer. All questionnaires were self-reported, written in English, and given as a hard copy distributed to participants by the research team.

The questionnaire was divided into five sections, as follows: (1) demographics; (2) depression, anxiety, and stress; (3) resilience; (4) psychological well-being; and (5) goal approach. Demographic questions included age, marital status, and academic year. The Depression and Anxiety Stress Scale (DASS-21) [26] was used to measure the levels of depression, anxiety, and stress. The DASS-21 contains 21 questions with subscales for each of the three domains. Each question has four answers, ranging from 0 “Did not apply to me at all” to 3 “Applied to me very much, or most of the time.” The score in each subscale ranged from 0 to 21, and the lower the score, the lower the level of psychological distress. The DASS-21 is a validated instrument in term of discriminant and convergent validity [27]. Also, it has good internal consistency as Cronbach’s alpha of 0.82 to 90 for each subscale [26].

Resilience was measured by the Resilience Scale (RS-14), which is a seven-point Likert scale [28]. Total scores range from 14 to 98, and the higher the score, the greater the resilience. RS-14 is a well-validated scale with a reliability of 0.91 [29]. Psychological well-being was measured with the Psychological Well-Being Scale–Short (PWB-S) [30–32], which is an 18-item seven-point Likert-type scale with responses ranging from 1, “strongly disagree,” to 7, “strongly agree.” It measures six psychological fields of well-being: autonomy, environmental mastery, self-acceptance, positive relations with others, purpose in life, and personal growth. Each one of them is calculated by sum field’s questions. PWB-S is not represented as one total score. Lower scores reflect low levels of psychological well-being [31]. This scale is validated with a Cronbach’s alpha of 0.84 [33]. Goal approach (the ability of participant to reach her goals) was assessed by asking the participants the following: “Please rate how close you feel right now to your goal of actually solving this problem,”, as this method to measure goal approach was taken from the previous study [34]. Participant responses were on a 0 to 10 scale, where 0 represented “not solved at all,” and 10 represented “completely solved.” All identifying data that were used to match participant’s identity for her data in T1 and T2 to were discarded after completing data collection and entry.

### Data analysis

SPSS version 21 software (IBM Corp., Armonk, NY, USA) was used as the data analysis tool. t-test was used to compare SG and CG at T1, then SG and CG at T2. Factorial repeated measure ANOVA (with partial eta squared) was used to assess the difference between SG and CG from T1 to T2 as within subject effect Also, paired t-test was used to assess the statistical changes in scores for each group separately, to assess clearly the difference occurred in time. Fisher’s exact test was used to assess the difference between SG and CG in term of the demographic data. a *P*-value < 0.05 was considered as significant level.

### Ethical approval and incentives

This study received prior ethical approval from UQU with the number 112-18. All of the participants signed a consent form before beginning the study. As an incentive, students who completed both questionnaires were randomly selected to win one of three SAR 100 vouchers good at a well-known Saudi Arabian bookstore.

## Results

**Fig. 2:**
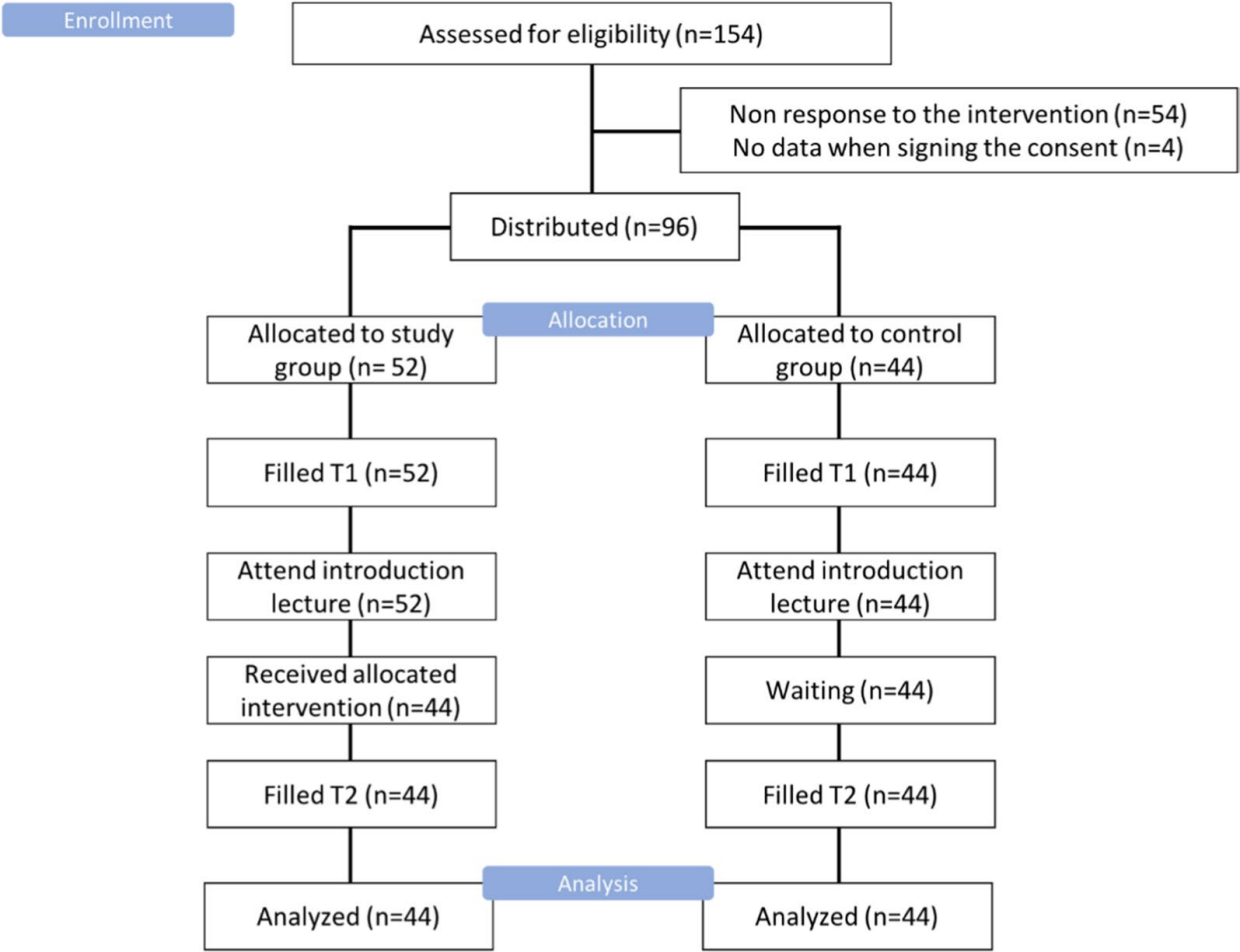
The pathway of the participants in the study

Out of 154 invited students, only 97 participated in the study (response rate = 63%). There were 52 participants in SG and 44 participants in CG. All of the students in the CG completed the T1 and T2 questionnaire, as shown in Fig. 2. However, there were nine participants who dropped out of the SG (drop-out rate = 10.2%). The mean (m) of participants’ age was 21.84, SD = 1.50, with a maximum of 24 and a minimum of 19. Other demographic data are displayed in Table 2. Fisher’s exact test and t-test results showed that the SG and the CG were not significantly different in terms of academic year, marital status, or age.

**Table 2:** Participant demographic data (n = 88)

Variable	Study Group (SG), *n* (%)	Control Group (CG), *n* (%)	Total, *n* (%)
Academic year			
2nd year	12 (13.64)	8 (9.09)	20 (22.72)
3rd year	3 (3.41)	3 (3.41)	6 (6.81)
4th year	4 (4.55)	13 (14.77)	17 (19.31)
5th year	12 (13.64)	9 (10.23)	21 (23.86)
6th year	13 (14.77)	11 (12.5)	24 (27.27)
Marital status			
Married	2 (2.27)	5 (5.68)	7 (8.00)
Unmarried	42 (47.73)	39 (44.32)	81 (92.00)

For the SG, a paired t-test was used to assess the difference in the outcomes between T1 and T2. The results showed that there were significant reductions in depression, anxiety, and stress and significant increases in resilience, self-acceptance as measured by the PWB-S scale, and goal approach. However, other PWB-S subscales were not significant, as shown in Table 3.

**Table 3:** Differences between the results of T1 and T2 in the study group

	Mean	SD	*P *value (paired t-test)	Cohen's d
Depression				
T1	6.84	4.74	0.002	0.416
T2	4.25	4.03		
Anxiety				
T1	6.57	4.34	0.044	0.232
T2	5.18	4.13		
Stress				
T1	10.09	3.81	< 0.000	0.525
T2	7.02	4.44		
Resilience				
T1	38.04	4.92	0.024	0.260
T2	39.95	5.46		
Autonomy				
T1	13.50	3.14	0.208	0.131
T2	14.05	2.79		
Environmental mastery				
T1	12.61	2.88	0.226	0.188
T3	13.39	2.98		
Personal growth				
T1	17.00	3.53	0.538	0.077
T2	17.36	3.10		
Positive relations with others				
T1	13.52	3.26	0.209	0.146
T2	14.23	3.61		
Purpose in life				
T1	15.32	2.79	0.670	0.056
T2	15.09	2.99		
Self-acceptance				
T1	14.48	3.28	0.001	0.305
T2	15.91	3.36		
Goal approach				
T1	4.14	2.30	< 0.001	0.762
T2	6.70	2.45		

In the CG, the results from a paired t-test showed that there was a significant reduction only on the RS-14 scale, whereas all other scales did not change significantly, as shown in Table 4.

**Table 4:** Differences between the results of T1 and T2 in the control group

	Mean	SD	*P *value (paired t-test)	Cohen's d
Depression				
T1	5.86	4.30	0.338	0.082
T2	5.36	4.32		
Anxiety				
T1	5.18	3.24	0.641	0.057
T2	4.91	3.46		
Stress				
T1	9.25	3.54	0.165	0.144
T2	8.45	4.30		
Resilience				
T1	37.49	4.77	0.006	0.246
T2	39.23	5.23		
Autonomy				
T1	14.82	2.55	0.753	0.030
T2	14.93	2.61		
Environmental mastery				
T1	12.84	3.05	1.000	0.000
T2	12.84	2.82		
Personal Growth				
T1	17.39	2.91	0.461	0.105
T2	16.95	3.03		
Positive relations with others				
T1	13.77	3.76	0.344	0.110
T2	13.14	4.31		
Purpose in life				
T1	14.70	3.28	0.441	0.107
T2	15.16	2.77		
Self-acceptance				
T1	15.02	3.43	0.789	0.027
T2	14.89	3.47		
Goal approach				
T1	4.20	2.18	0.318	0.073
T2	4.43	2.29		

To compare the differences in the tested scales from T1 to T2 between the SG and the CG, factorial repeated ANOVA was conducted to compare between the SG and CG with the interaction of time (as within subject effect). Effect size was represented as partial eta square. The results showed that there were significant differences in the depression, stress, self-acceptance, and goal approach measurements. Conversely, the other measurements showed no significant differences. The results are shown in Table 5.

**Table 5:** Factorial repeated ANOVA to compare the SG and CG with the interaction of time (within subject effect)

Area MEASURED	df(F)	*p *value	Partial eta square
Depression	1(5.12)	0.026	0.0256
Anxiety	1(1.578)	0.212	0.018
Stress	1(6.892)	0.01	0.074
Resilience	1(0.026)	0.872	0
Autonomy	1(0.599)	0.441	0.007
Environmental mastery	1(0.893)	0.347	0.1
Personal growth	1(0.930)	0.338	0.11
Positive relations with others	1(2.404)	0.125	0.009
Purpose in life	1(0.749)	0.389	0.027
Self-acceptance	1(5.660)	0.02	0.062
Goal approach	1(86.000)	< 0.001	0.226

Partial eta square: (small effect size = 0.01, medium effect size = 0.06, large effect size = 0.15) [43]

## Discussion

Using an interventional design, this study aimed to assess the impact of life coaching on female dental students’ psychological health. The results indicated significant reductions in students’ levels of depression and stress, and significant elevations in the levels of self-acceptance and goal approach as compared to the CG.

When we compared our study with previous studies, we found some similarities and some differences. For example, the Australian study by Grant [34] indicated that solution-focused coaching resulted in an improvement in psychological health and goal approach. This was similar to our findings; both studies used solution-focused life coaching and made the post-intervention measurement immediately after the last coaching session. The two studies differed in that the Australian study was an RCT conducted with a larger sample size, and the participants were psychology students rather than dental students.

The only prior study conducted with dental students in Saudi Arabia [11] indicated that a self-development coaching program reduced levels of depression and anxiety as compared to a control group. This is similar to our study in some aspects, as both studies resulted in a reduction in depression levels. However, our study also resulted in a reduction of stress levels, while the previous study showed a reduction in the levels of anxiety. This difference might be due to the difference between the two interventional studies given that the previous study by Aboalshamat et al. [11] was a short intervention of two days using sessions that involved a different style of coaching. The prior study coaching involved one coach giving information, along with motivation, to a gathering of participants who were then expected to implement the information on their own. Our study, however, was conducted over 6 weeks using a conventional solution of focused life coaching [23, 34] using one-on-one sessions given by different coaches.

In terms of our study’s limitations in comparison to Aboalshamat et al. [11], because that study used an RCT with a larger sample size of both males and females and had a longer follow-up assessment period, it is a higher quality study. Nonetheless, our results combined with those of the prior study indicate that coaching in general has a positive impact on psychological health, but the different coaching styles and durations of coaching might result in different improvements in different aspects, as found by another study [34]. Further research is needed that should be conducted with solution-focused life coaching among dental students (both male and female) using an RCT design and a longer follow-up period in order to validate our results.

The German RCT study [20] found a significant reduction in stress after a two-session psycho-educative seminar in comparison to a control group, but there were no significant changes in depression or anxiety. This is similar to our findings in regard to stress, but our study also showed improvement in depression. This might be due to the difference in the coaching program itself and/or the duration of the intervention, as previously noted.

The last study to compare is the pilot interventional study using the Physician Well-being Coaching program with 11 physicians, which resulted in an increase of participants’ resilience via improvement in self-awareness, self-care, self- compassion, prioritization, and boundary setting [19]. Our study also found an increase in resilience after the intervention for the SG. Nevertheless, this improvement was not statistically significant in comparison to the CG. This explains the differences between the two studies’ conclusions. In fact, the previous study by Schneider et al. [19] used a different coaching program, with fewer coaching sessions, but over a longer period. They also used qualitative assessment. This actually highlights the importance of having a control group included in the study design to eliminate the possibility of deceptive results in improvement, especially with psychological studies [35, 36].

Per these comparisons of studies, it seems that coaching program content and implementation, duration of coaching sessions, and number of coaches might influence the outcomes of coaching studies. This conclusion was also reached in one of the prior studies [11]. Also, coaching in general seems to be effective for improving psychological aspects in both medical and dental students or practitioners and do so in a similar pattern. However, we cannot validate our claims without further research.

According to our findings, we recommend offering coaching sessions to dental students to help them to cope with their heavy psychological burden. Students who have been formally trained in the technique could offer these coaching sessions, which seems to be feasible and convenient, as conveyed in our study. Our findings indicate that a 15 min coaching session on a weekly basis may be effective, and this period seems reasonable from effort and time points of view. However, it is important to note that coaching may not be the appropriate choice of psychological modality for clinical psychological conditions [37], such as suicidal ideation or attempts. Also, it is recommended that future studies use RCT, have a longer follow-up period, and use a larger sample size that includes both genders in order to obtain more generalizable results about the effectiveness of coaching. It should be noted that our study was conducted only with females, and male students may behave differently, as females tend to be more emotional [38] and have greater emotional intelligence than males [39]. Most importantly, due to a lack of studies with evidence-based approaches in the literature, more interventional studies are needed to address the psychological burden faced by dental students and provide proper evidence-based approaches to help improve their mental health [11]. Also, this study supports a recent study that encouraged train medical students in coaching to help them to have solution-focused mindset [40].

This study had a number of limitations, including a lack of randomization, as this is a quasi-experiment, which increases the potentiality of selection bias. Participants who selected to be in the SG might have different characteristics than those in the CG, which urge us for further study using randomized control trial stud design, which was not possible in this study. Other limitations include individual differences in the coaches and their personalities, having only female dental student participants, and having no long-term effect assessment. However, this study utilized validated instruments, acceptable drop-out levels (did not exceed 20%) [41, 42], and the CG for comparison. We also used a standardized life coaching program [23] that can be applied by other researchers. To the best of our knowledge, this is the first study conducted in Saudi Arabia using a life coaching program as an intervention.

## Conclusions

The solution-focused life coaching approach used in this study seems to be effective for reducing psychological burden and mental health problems among dental students. In particular, the coaching was found to be effective in the aspects of depression, stress, and self-acceptance, but it was not statistically significantly different in other psychological aspects, such as anxiety, resilience, autonomy, environmental mastery, personal growth, positive relations with others, and purpose in life. The coaching was also found to be effective for improving dental students’ goal approaches. We recommend that coaching be used as an institutional method to help dental students cope with their psychological problems.

## Data Availability

The data file analyzed for this study is available upon reasonable request.

## Abbreviations

DASS-21: Depression and Anxiety Stress Scale
RS-14: Resilience Scale
PWB-S: Psychological Well-Being Scale–Short
T1: Baseline questionnaire completed before the intervention
T2: Follow-up questionnaire completed after the intervention
SG: Study group
CG: Control group
UQU: Umm Al-Qura University
SMART: Specific, measurable, achievable, realistic, and time
m: Mean
SD: Standard deviation
df: Degree of freedom
